# Brain Structure Alterations in Poly-Drug Use: Reduced Cortical Thickness and White Matter Impairments in Regions Associated With Affective, Cognitive, and Motor Functions

**DOI:** 10.3389/fpsyt.2019.00667

**Published:** 2019-09-20

**Authors:** Human F. Unterrainer, Michaela Hiebler-Ragger, Karl Koschutnig, Jürgen Fuchshuber, Klemens Ragger, Corinna M. Perchtold, Ilona Papousek, Elisabeth M. Weiss, Andreas Fink

**Affiliations:** ^1^Center for Integrative Addiction Research (CIAR), Grüner Kreis Society, Vienna, Austria; ^2^University Clinic for Psychiatry and Psychotherapeutic Medicine, Medical University Graz, Graz, Austria; ^3^Institute for Religious Studies, University of Vienna, Vienna, Austria; ^4^Institute of Psychology, University of Graz, Graz, Austria

**Keywords:** DTI, gray matter, neuroplasticity, poly-drug use, VBM, white matter

## Abstract

Substance use disorders (SUDs) are defined by obsessive and uncontrolled consumption, which is related to neurobiological changes. Based on previous work, this study investigated potential alterations in brain structure in poly-drug use disordered (PUD) patients in comparison to controls from the normal population. This study involved a sample of 153 right-handed men aged between 18 and 41 years, comprising a clinical group of 78 PUD and a group of 75 healthy controls. Group differences in gray matter (GM) and white matter (WM), as well as cortical thickness (CT), were investigated by means of diffusion tensor imaging using automated fiber quantification (AFQ) and voxel-based morphometry. We observed significant WM impairments in PUD, especially in the bilateral corticospinal tracts and the inferior longitudinal fasciculi. Furthermore, we found reduced CT in the PUD group especially in the left insular and left lateral orbitofrontal cortex. There were no group differences in GM. In addition, PUD exhibited a higher amount of psychiatric symptoms (Brief Symptom Inventory) and impairments in cognitive functions (Wonderlic Personnel Test). In line with previous research, this study revealed substantial impairments in brain structure in the PUD group in areas linked with affective, cognitive, and motor functions. We therefore hypothesize a neurologically informed treatment approach for SUD. Future studies should consequently explore a potential positive neuroplasticity in relation to a better therapeutic outcome.

## Introduction

Within the European Union, a lifetime prevalence of up to 3% for substance use disorders (SUDs) has been shown for the general population (1, 2). Correspondingly, SUD represent a significant burden on society and healthcare systems. In addition to this, the treatment of SUD has been reported to be extremely difficult due to a high proportion of therapy dropouts (3). SUD have been most prominently described as a chronic, relapsing brain disorder characterized by compulsive drug use, which produces long-term changes in the reward circuitry of the brain (4–6). Therefore, it is now widely accepted that many drugs may “hijack” the reward centers of the brain, setting in motion a downward spiral towards SUD (7). Notably, some authors have challenged this view by arguing that the complex mechanisms underlying SUD cannot be explained by neural dysfunction alone (8). In that sense, SUD have also been widely discussed in relation to dysfunctional attempts of self-medication (9) and misled attachment needs (10). Furthermore, it should be noted that premorbid brain abnormalities might also lead to severe psychiatric disturbances such as SUD (11, 12).

From a developmental perspective, childhood and adolescence represent critical periods of cortical development related to lifelong adult characteristics. This development is likely interrupted by drug misuse since most people usually start abusing drugs in puberty (13). Although acute drug intake increases dopamine neurotransmission, chronic drug consumption results in a significant decline of dopamine activity, associated with, among other things, dysregulation of the orbitofrontal cortex and the cingulate gyrus (14), which in turn is linked to maladaptive decision making (15) and increased drug craving (16) in SUD. However, because SUD patients usually show a more or less haphazard kind of poly-drug use, it is as yet largely unclear which detrimental effects are caused by the abuse of which drug (17, 18). In addition, all drugs have similar direct or indirect effects on the mesolimbic reward system. This system extends from the ventral tegmentum to the nucleus accumbens and projects to areas such as the limbic system and the orbitofrontal cortex (6).

Furthermore, different kinds of drugs have been observed as being associated with impairments of white matter (WM) (19) as well as gray matter (GM) (20, 21) in the brain. Here, a special focus has been placed on the detrimental effects of drug use (especially cannabis) on brain structure and functioning in adults and adolescents (22, 23). There is substantial evidence that heavy substance abuse might be particularly harmful to the development of WM during adolescence (19, 24–27). Correspondingly, cognitive deficits were reported in a group of methamphetamine users, which in turn were related to lower whole-brain cortical thickness (CT) (12). Therefore, chronic drug use might cause deficits in and/or a failure to develop normative cognitive abilities (12).

Numerous studies on SUD have observed positive as well as negative neuroplasticity, which generally means the alteration of the brain’s structure, as the result of various learning templates (28–30). This is consistent with the assumption that SUD represent a pathological but powerful form of learning and memory (31). Notably, numerous studies on structural neural parameters in SUD has shown impairments in various networks of the brain (Hiebler-Ragger et al., submitted, 32–34), in particular those linked with frontal volitional control and the reward-salience centers (35). In correspondence to this, recovery from SUD was observed to correlate with positive neuroplasticity, such as the return to more gyral volumes (36) and enlargement of GM after mindfulness therapy (37). Taken together, a broad knowledge of the neurobiological alterations linked with SUD, along with the brain networks associated with successful abstinence, can hence improve our understanding of SUD and its treatment in general (17).

To date, most of the research in this area has investigated the role of WM, GM, or CT and their relation to SUD independently from one another, resulting in a rather isolated picture of findings on structural brain deficits in poly-drug use disorder (PUD). Therefore, the goal of the present study was a comprehensive investigation of potential differences in WM, GM, and CT in a large sample of PUD patients compared to healthy controls. Following recent developments in the field of WM analysis, we used automated fiber quantification (AFQ) for a more detailed assessment of WM differences between PUD patients and controls. The particular strength of this study could be seen in the application of a multimodal imaging approach, assessing different characteristics of brain structure and related functions within one and the same sample of participants. Such an approach is particularly motivated by the fact that different characteristics of GM or WM morphology (such as CT, GM volume, or myelination), each of them subserving different cognitive, affective, and motor functions, may be affected in PUD in different ways. On the basis of previous work indicating that heavy substance abuse might be particularly harmful to the development of WM during adolescence (19, 24–27), and on the basis of our previous studies with PUD patients (33, 34), we expected substantial differences especially in WM integrity between PUD patients and the control group from the normal population. Available evidence (12, 20, 21) leads us to assume that different parameters of GM morphology (volume and CT) are affected as well.

## Methods and Materials

### Participants

A total sample of 153 right-handed men between 18 and 41 years of age, composed of one clinical and one nonclinical group, was investigated. This sample integrated data from three different studies related to other research questions regarding PUD (Hiebler-Ragger et al., submitted, 33, 34). In detail, 45 participants (PUD patients: *n =* 29) were included from the first study (34) that focused on WM integrity in relation to attachment and personality. Sixty-five participants (PUD patients: *n =* 25) were included from the second study (33) that focused on WM integrity in relation to negative affective states, and 43 participants (PUD patients: *n =* 24) were included from the second study (Hiebler-Ragger et al., submitted) that focused on neural activation during emotion regulation efforts. Data acquisition took place over a time span of 4 years, starting in January 2014 and ending in November 2016. The clinical group (*n =* 78) was diagnosed for PUD (F19.2) by a licensed psychiatrist (a medical doctor specialized in psychiatry with 20 years of experience of treating SUD patients) according to the International Classification of Diseases version 10 (38). The nonclinical comparison group was comprised of students from various faculties (CG; *n =* 75). Students were included in the nonclinical groups if they were free from any past or present psychiatric disorder or chronic disease. With regards to the use of psychotropic substances, CG included 47 nonsmoking students who reported either no experience with illegal substances or to have tried them just a few times in their life, as well as 28 nicotine smoking students who reported using illegal substances primarily for recreation at least once a week during the last month. Psychometric assessment of the clinical participants took place in two therapeutic facilities of the “Grüner Kreis” society, where these participants were undergoing long-term SUD treatment based on the “Therapeutic Community” concept (39). The “Grüner Kreis” society (founded in 1983) is Austria’s biggest institution for long-term drug therapy. Usually, the patients stay from 6 to 18 months within the Therapeutic Community. All behavioral assessments were conducted *via* group testing. Participants’ consent was obtained according to the Declaration of Helsinki. Individuals were only included in the study if they did not report general MRI contraindications (e.g., head injuries, metal implants), major physical disorders, or severe cognitive impairments including acute psychotic episodes. The study was approved by the authorized ethics committee. See **Table 1** for detailed demographic information.

**Table 1:** Group differences (ANOVAs) in demographics and behavioral measures.

Measure	α	CG (*n* = 75)		PUD (*n* = 78)		F_(_ *_1,34_*_)_	*η*²
		*M*	*SD*	*M*	*SD*		
Age	–	25.28	3.37	28.71	5.15	23.48**	0.14
Education (years)	–	13.92	2.82	11.51	2.58	30.39**	0.17
Treatment (weeks)	–	–	–	24.88	18.46	–	–
**WPT**	–	28.95	6.02	17.51	7.16	113.89**	0.43
**BSI**							
GSI	0.88	10.32	7.66	15.47	11.01	11.22**	0.07
Anxiety	0.73	4.67	3.56	5.85	3.92	3.79	0.02
Depression	0.78	3.45	3.67	6.08	4.67	15.79**	0.10
Somatization	0.72	2.22	2.77	3.55	3.94	5.79*	0.04

*p < 0.05, ** p < 0.01, CG , control group; PUD , poly-drug users; BIS, Brief Symptom Inventory; GSI, Global Severity Index; WPT, Wonderlic Personnel Test.

### MRI Acquisition

Imaging data were acquired on a 3T Siemens Skyra (Siemens Healtheneers, Erlangen, Germany) with a 32-channel head coil. Since the sample of this study consists of three different studies, two different sequence protocols were used, with slight variations in sequencing parameters. For all participants, T1-weighted images as well as diffusion-weighted images were acquired. Details of imaging parameters are itemized in **Table 2**.

**Table 2:** Details of imaging parameters.

	T1		Diffusion tensor imaging (DTI)	
	Study 1 & 2	Study 3	Study 1 & 2	Study 3
	44	100	44	100
TR (repetition time, ms)	2,300	1,680	8,500	3,036
TE (echo time, ms)	2.96	1.89	83	104.6
TI (inversion time, ms)	900	1,000	–	–
FoV (field of view, mm)	256	224	256	240
Slices (#)	176	192	64	66
Slice—thickness (mm)	1.2	0.88	2	2.5
Gap (mm)	0.5	0.44	0	0
Matr.size	256	256	128	96
Flip angle (°)	9	8	90	86
Voxel (mm)	1 iso	0.88 iso	2 iso	2.5 iso
Directions	–	–	64	64
PAT (Parallel Acquisition Techniques)	0	0	Grappa	Multiband factor = 3
*b* value	–	–	1,000	2,000
Reverse b0	–	–	No	Yes

### MRI Data Preprocessing and Analysis

#### Diffusion

Data preprocessing was performed using the software package MRtrix (40) and FSL (41). First, data were visually inspected for artifacts and then denoised with the MRtrix command “dwidenoise” (42). Estimation and correction of geometric distortion was carried out with FSL’s “top up” and “eddy” using the nondiffusion-weighted images (*b* value = 0) collected with reverse-phase encoding direction (43). Datasets with no reverse encoding direction image available were corrected with eddy_correct. Next, individual B0 images were coregistered to the structural image using SPM12 (v7219; Wellcome Trust Centre for Neuroimaging). The coregistered T1-images were then segmented into five tissues using the “5ttgen” algorithm (44). This step is necessary to allow the estimation of the response function for each tissue-class separately. The response function was estimated for GM, WM, and cerebrospinal fluid. Fiber orientation distributions (FODs) were computed using these multitissue-constrained spherical deconvolutions (45). FODs were then used to compute whole-brain fiber tractography with 5 million tracks. As a last preprocessing step, the scale-invariant feature transform algorithm was used to reduce tractogram biases (46) reducing the number of tracks to 1 million.

#### Tract Quantification

Whole-brain tractography data were imported into the AFQ software package (https://github.jyeatman/AFQ) (47) running on MATLAB (2017b, The Mathworks, Natick, MA, USA), which identifies 20 major fiber tracts, including the right and left thalamic radiations, forceps major and minor of corpus callosum, right and left inferior fronto-occipital, inferior longitudinal, arcuate and uncinate fasciculi, corticospinal tract, and cingulum. To assess differences in tensor-based indices along each pathway, whole-brain tractography was normalized into the MNI space, and each fiber pathway was evenly spaced into 100 cross-sectional nodes. The mean fractional anisotropy (FA) in each node was calculated, and group differences were analyzed for each node within each pathway. Multiple comparison corrections were conducted using the AFQ software package script AFQ_MultiCompCorrection.m, which is based on Nichols and Holmes (48). Using this script, the family wise error corrected alpha value for pointwise comparison was computed for each tract to correct for multiple comparison. As a result, *p* values below a threshold of <0.0025 (0.05/20 tracts) were considered significant.

#### Voxel-Based Morphometry

Structural scans were analyzed using the Computational Anatomy Toolbox (CAT12; r 1274) implemented in SPM12, running under Matlab 2017b, to assess voxel-wise comparison of GM volume (GMV) differences. Data were visually checked and the segmented, modulated, and normalized into the MNI space (1.5mm). The sample homogeneity was checked, and the total intracranial volume (TIV) was estimated. Finally, data were smoothed with a Gaussian kernel with a full width at half maximum of 8 mm.

#### Cortical Thickness

The CAT12 toolbox was again used to extract CT. This fully automated method uses tissue segmentation as already done in the voxel-based morphometry (VBM) analysis and uses a projection-based algorithm to compute CT (49). Finally, surface data were smoothed with a Gaussian kernel with a full width at half maximum of 15 mm.

For statistical analysis of GMV and CT parametric-free permutation tests (TFCE toolbox, number of permutations = 10,000) were used. Age and TIV (only VBM) were included in the statistical model as regressors of no interest. Results were considered statistically significant with *p* < 0.05 corrected for family-wise error.

### Behavioral Measures

#### Psychiatric Symptoms

The Brief Symptom Inventory-18 (BSI-18) (German adaptation by (50) is a short version of the highly established Symptom Checklist SCL-90-R (51). The amount of psychiatric burden for the preceding 7 days for three dimensions of psychiatric symptoms (Somatization, Depressiveness, and Anxiety) is assessed by means of 18 items (6 items for each subscale). The BSI-18 employs a 5-point rating form ranging from 1 (absolutely not) to 5 (very strong). It is also possible to sum up the 18 items into a total score: The Global Severity Index (GSI) of psychiatric symptoms. In previous research, Cronbach’s alpha was observed to be at least 0.79 for all the subdimensions (33). See **Table 1** for details.

#### Cognitive Ability

Participants also completed the Wonderlic Personnel Test (WPT), a rough screening instrument for the assessment of intelligence (52). This test requires the processing of disordered sentences, analogies, number series, word and sentence comparisons, and geometrical figures within a given time period of 12 min. The WPT contains 50 items with increasing difficulty. The total score is generated from the number of correct responses. See **Table 1** for details.

#### Behavioral Data Analysis

For group comparisons, one-way analyses of variance were conducted. Post hoc comparisons were conducted with Tukey’s honest significant difference test. Pearson`s correlations were calculated to investigate the relationship between neural and behavioral parameters. Alpha was set to *p* < 0.05. Eta squared (*η* ^2^) is given as estimate of effect sizes.

#### Data Availability

The datasets generated during and/or analyzed during the current study are available from the corresponding author on reasonable request.

## Results

### Demographics and Clinical Characteristics

As shown in **Table 1**, PUD patients were older than the controls (PUD: *M* = 28.71, SD = 5.15; CG: *M* = 25.28, SD = 3.37; *p* < 0.001; *η*² = 0.14). Analyses also revealed significant differences in education, with the CG reporting an average of 14 years (SD = 2.82) of education, whereas the PUD patients’ average was 12 years (SD = 2.58) of education (*p* < 0.001; *η*² = 0.17). At the time of data acquisition, the PUD patients were undergoing inpatient SUD treatment within a therapeutic community for a mean time of 25 weeks (SD = 18.46). They reported a history of drug abuse over an average period of 12 years (SD = 5.57; range, 2–27 years; missing values, 24). Forty-eight PUD patients were undergoing maintenance therapy, while 30 PUD participants reported living in abstinence. Fifty-three PUD patients received psychopharmacological medication (antidepressant: *n* = 20; antipsychotic: *n* = 23; anxiolytic: *n* = 5; other: *n* = 20).

PUD exhibited a significantly higher amount of Depressiveness (*p* < 0.01; *η*² = 0.10), Somatization (*p* < 0.05; *η*² = 0.04) as well as a higher score for the total Global Severity Index in the BSI-18 (*p* < 0.01; *η*² = 0.07). Accordingly, PUD patients showed no differences when compared to normative data for psychiatric inpatients (50), while CG participants exhibited less Depressiveness and a lower score in the Global Severity Index (for both *p* < 0.01). The intercorrelations between demographic and behavioral parameters in PUD can be retrieved from **Table 3**. Age was positively related to cognitive abilities (*p* < 0.01) as well as depression (*p* < 0.05) and the duration of treatment (*p* < 0.05).

**Table 3:** Correlations between demographic and behavioral characteristics in PUD (n = 78).

	1.	2.	3.	4.	5.	6.	7.	8.
1. Age	–	0.22	0.30**	0.14	0.24*	0.06	0.17	0.25*
2. Education (years)		–	0.20	0.17	0.20	0.04	0.16	0.08
3. WPT			–	0.04	0.02	−0.05	0.01	0.19
**BSI**								
4. Anxiety				–	0.68**	0.67**	0.88**	0.14
5. Depression					–	0.63**	0.89**	0.11
6. Somatization						–	0.86**	0.07
7. GSI							–	0.12
8. Treatment								–

*p < 0.05, **p < 0.01, PUD, poly-drug users; WPT, Wonderlic Personnel Test; BIS, Brief Symptom Inventory; GSI, Global Severity Index; Treatment, treatment duration.

### Differences in White Matter, Gray Matter, and Cortical Thickness Between PUD and Controls

#### White Matter Fiber Tracts

**Figure 1:**
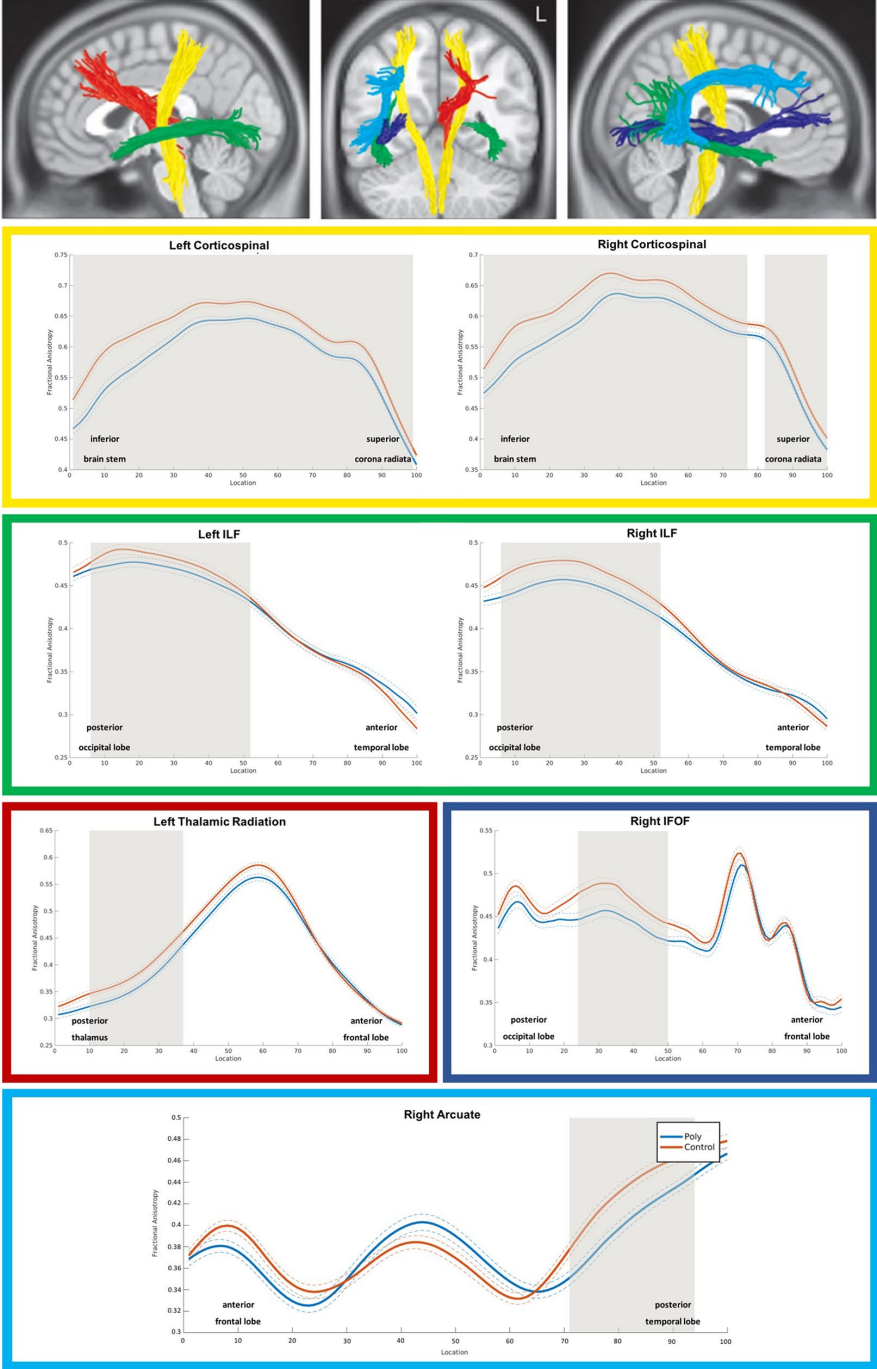
3D visualization and tract diffusion profiles for white matter fiber tracts showing significant differences between PUD and controls. Note. Yellow = left and right corticospinal tract; green = left and right inferiore longitudinale fasciculus (ILF); red = left thalamic radiation; blue = right inferior fronto-occipital fasciculus IFOF; cyan = right arcuate fasciculus; shades of gray in the profiles indicate nodes with significant group differences. PUD, patients with poly-drug use disorder.

As shown in **Figure 1**, PUD patients exhibited significant reductions in FA relative to controls across the entire left and the majority of nodes of the right corticospinal tract. In addition, there were significant FA reductions in posterior portions of the bilateral inferior longitudinal fasciculi and in smaller portions of the left thalamic radiation, the right inferior fronto-occipital fasciculus, and the right arcuate fasciculus.

#### Gray Matter Volume

Voxel-based morphometry analyses revealed no significant differences in GMV between PUD and controls.

#### Cortical Thickness

**Figure 2:**
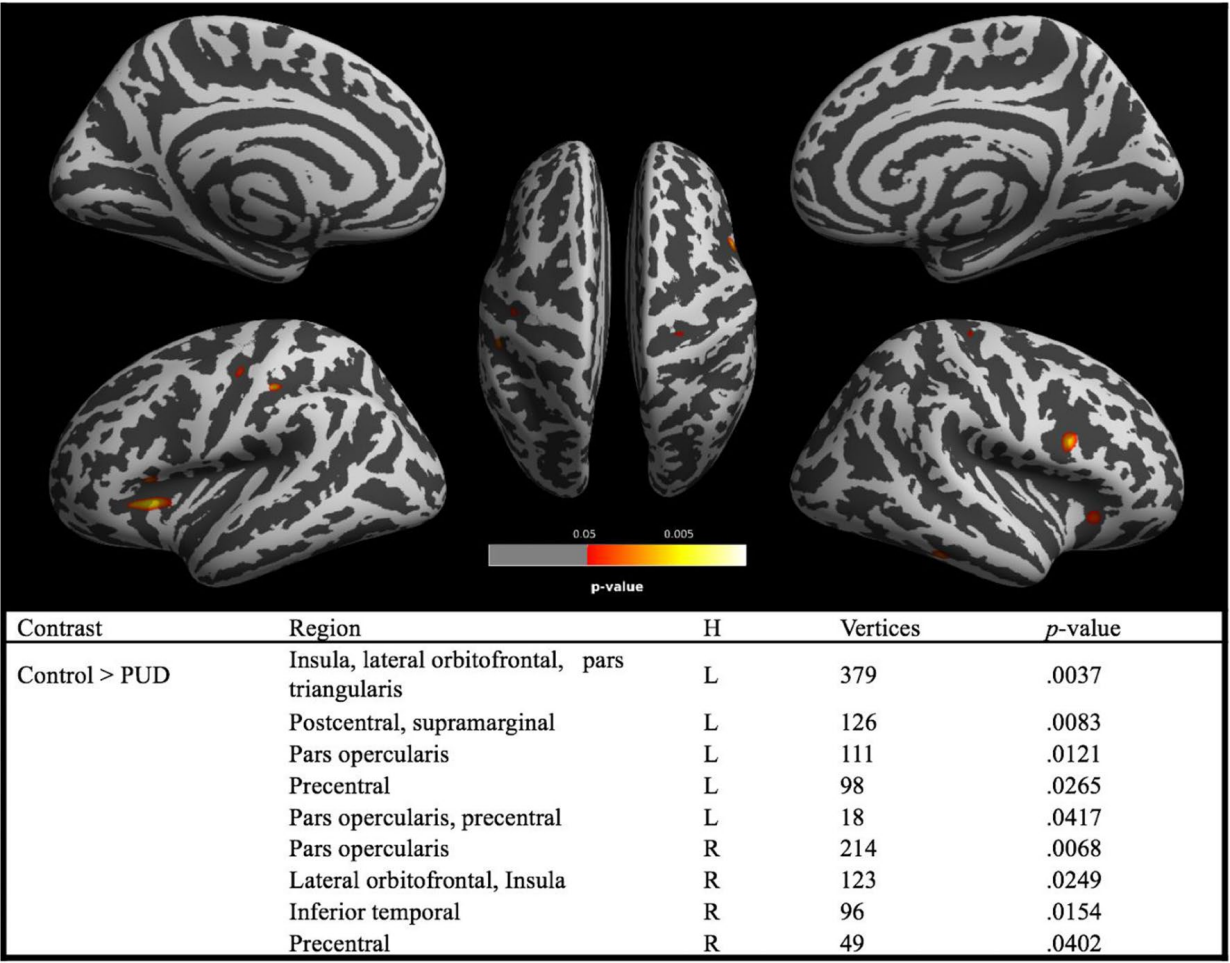
Brain regions with significant (red–yellow) group differences in cortical thickness between PUD and controls. *Note*. PUD, poly-drug users; H, hemisphere; L, left; R, right; brain regions are derived from Desikan–Killiany DK40 Atlas. Reported *p* values are TFCE corrected for family-wise error (*p* < 0.05).

Analyses revealed brain regions with significant reductions of CT in PUD relative to controls, while there were no brain regions with higher CT in the patient group (see **Figure 2**). The largest cluster comprised the left insular and the left lateral orbitofrontal cortex. There were also significant CT reductions in the right orbitofrontal cortex. Generally, as is the case for the lateral orbitofrontal cortex, CT reductions were bilateral. This particularly applies to regions of the inferior frontal gyri (pars opercularis) and the precentral gyri. In addition, analyses revealed CT reductions in a cluster involving the left postcentral gyrus and small portions of the supramarginal gyrus in addition to a cluster in the right inferior temporal lobe.

## Discussion

This study investigated alterations in brain structure in an unprecedented large sample of PUD patients compared to controls from the general population. Analyses revealed impaired WM integrity along with reduced CT in the PUD sample but no alterations in GM. These findings were mirrored by significant differences between PUD and healthy controls regarding behavioral measures, such as a higher amount of psychiatric symptom burden as well as lower cognitive abilities. Furthermore, our results confirm previous research indicating substantial deficits especially in WM circuitry in PUD patients (25, 26, 33, 34, 53).

Deficits in WM structure might represent a valid predictor for negative therapeutic outcome. For instance, Moeller et al. (44) reported that deficits in WM integrity are related to an increased amount of impulsivity in cocaine-dependent patients. A high amount of impulsivity has been widely shown as being a risk factor for the development of SUD (54) as well as a substantial predictor for a negative SUD therapy outcome (55). Furthermore, we observed lower CT in PUD patients, which has been linked to higher memory deficits (12) as well as reduced effortful attention performance (56). CT abnormalities have been observed to be associated with SUD such as alcohol dependence (57), marijuana misuse (58), and nicotine smoking (59), as well as nonsubstance-related disorders such as excessive internet use (60) and online gaming (61). Moreover, significant abnormalities in CT were reported in individuals with heavy prenatal alcohol exposure. These abnormalities were found to be linked with impairments in verbal recall and visuospatial dysfunction (62).

Notably, we did not find any significant differences in GM between PUD patients and healthy controls. This is in clear contrast to previous research, where disrupted GM was observed to be related with several SUD, for example alcoholism (63), cocaine use (20), and cannabis use (23). We interpret our conflicting findings as showing that, in our rather young PUD sample, WM paths might be among the first to become affected by PUD. In fact, previous work suggested that heavy substance abuse might be particularly harmful to the development of WM during adolescence (19, 24–27). In this regard, it is conceivable that, in its earlier stages, PUD already compromises more basic “hardware” processes, e.g., motor functions as indicated by the substantial WM deficits in the bilateral corticospinal tract, while further impairments in higher order cognitive functions only result after prolonged consumption. Accordingly, at this point, we can only assume that, later in life, GM might become damaged, too. For instance, in a study of Qiu et al. (64), the mean age of the group of heroin dependents was considerably higher (*M =* 35 years, SD *=* 4.2). Here, the authors reported a progressive deterioration of WM microstructure dependent on the duration of heroin use.

From a developmental perspective, the finding of diminished CT in PUD fits nicely with the literature. For instance, Hilton Jr (29) described lower CT as a kind of premorbid cortical weakness, which leads to poor cognitive performance and could pave the way to develop a SUD later in life. In support of this notion, we observed a significantly lower level of cognitive ability, along with a lower educational status in the group of PUD patients. Even more importantly, in PUD patients, reduced CT, especially in regions of the insular and the orbitofrontal cortex, may suggest that these structural alterations mirror difficulties in affective processing, specifically emotional awareness (65) and emotional regulation (66), which are known to be compromised in SUD (67–69). This is further supported by the fact that we found pronounced WM impairments in the bilateral inferior longitudinal fasciculi and in the right inferior fronto-occipital fasciculus, which are both known as key components of a face processing network (70), with an important role in facilitating the ability to discriminate between emotional expressions in faces (71). In accordance with the general notion of impaired affective processing and emotion regulation in PUD, we previously observed a significantly reduced capacity for using cognitive reappraisal to regulate anger in PUD patients when compared to controls from the normal population (Hiebler-Ragger et al., submitted).

According to Kalivas and O’Brien (72), SUD is based on pathological changes in brain function, which are produced by a repeated pharmacological assault on the brain circuits that regulate how a person behaviorally responds to certain stimuli. Since recovery from SUD has been correlated with positive neuroplastic changes (36), neuroplasticity might therefore constitute a highly important indicator for the evaluation of therapeutic outcome. Especially for long-term treatment of SUD, neurologically informed therapeutic interventions may represent an important resource (33). Changes in cognitive and affective abilities in SUD patients during long-term treatment might be intertwined with neuroplastic effects (5, 10). Strikingly, (73) reported beneficial effects of transcranial stimulation (TMS) for the treatment of SUD, as TMS seems to facilitate long-term neurophysiological changes which have the potential to affect behaviors relating to drug craving, intake and relapse. Accordingly, a respective research focus on neuroplasticity in SUD patients may provide additional valuable information for the clinical outcome evaluation.

## Limitations and Future Perspectives

The study of PUD populations has previously been discussed as being too unspecific (18). However, as a counter argument, from a clinical perspective, it is evident that a high rate of SUD patients is diagnosed with PUD because of a completely chaotic pattern of consumption (74). In this study, we did not further control for maintenance therapy since previous research revealed no differences in neural and behavioral parameters between PUD patients in maintenance therapy and abstinent patients (34). Furthermore, there was no perfect age match between the two groups, as the healthy control group was significantly younger than the SUD patients [3.43 years (see **Table 1**)]. However, we observed age to be weakly related with behavioral characteristics, such as Intelligence, Depression, and Duration of Treatment (**Table 3**). In addition, in the analysis of GM and CT, age was considered as a regressor of no interest in the statistical model. Additionally, in future research, potential gender differences might be considered another factor of study, as, for instance, Sawyer et al. (75) reported sex differences in alcoholism-related abnormalities of WM connectivity. Furthermore, in this study, we sought to focus primarily on potential differences between PUD patients and a nonsubstance use disordered control sample regarding the areas of WM and GM as well as CT, based on an enhanced sample. In further analysis, we intend to investigate potential connections between neural parameters and an extended set of behavioral parameters in PUD more in detail, which might reveal further insights concerning individual differences in PUD. These findings will be published somewhere else. The cross-sectional design in this study limits the possibilities of interpretation. Thus, we can only speculate on the causal relationship between impairments in brain structure and the development of PUD as well as on potential neuroplastic effects during PUD treatment. While the combined analyses of several neural and behavioral parameters in the rather large sample of this study reveals important insights into the clinical profile of PUD patients, a longitudinal research approach comprised of several measurement points is highly warranted in order to be able to say more about the clinical relevance of neuroplasticity for patient treatment.

## Ethics Statement

This study was carried out in accordance with the recommendations of Ethic guidelines, Ethics Board, University of Graz; with written informed consent from all subjects. All subjects gave written informed consent in accordance with the Declaration of Helsinki. The protocol was approved by the Ethics board, University of Graz.

## Author Contributions

HU and AF conceptualized the study. MH-R, JF, KK, KR, CP, HU, and AF collected, analyzed, and interpreted the data. HU and MH-R drafted the manuscript. CP, IP, EW, and AF critically reviewed the manuscript. All authors gave their final approval of the manuscript.

## Conflict of Interest Statement

The authors declare that the research was conducted in the absence of any commercial or financial relationships that could be construed as a potential conflict of interest.
